# The Takotsubo Syndrome: Clinical Diagnosis Using POCUS 

**DOI:** 10.24908/pocus.v7i1.15296

**Published:** 2022-04-21

**Authors:** Josu López Libano, Lorenzo Alomar Lladó, Leire Zarraga López

**Affiliations:** 1 Intensive Care Unit, Juaneda Miramar Hospital Palma de Mallorca Spain; 2 University of the Basque Country, UPV/EHU UPV/EHU

**Keywords:** Takotsubo, Point of Care Ultrasound (POCUS), systolic anterior movement of the mitral valve (SAM)

## Abstract

Takotsubo syndrome is a cardiomyopathy that can mimic an acute heart attack, in terms of clinical presentation, electrocardiographic changes, and findings on echocardiogram. Point-of-care-ultrasound (POCUS) can be used to detect this condition, even though the definitive diagnosis is made angiographically. We present the case of an 84-year-old woman with a subacute coronary syndrome and high levels of myocardial ischemia markers. The POCUS performed on admission showed characteristic left ventricular dysfunction involving the apex but sparing the base. The coronary angiography ruled out significant arteriosclerotic in the coronary arteries. The wall motion abnormalities were partially corrected in the 48 hours after admission. POCUS might be a useful tool to establish an early diagnosis of Takotsubo syndrome at time of admission.

## Clinical Case 

**Figure 1:**
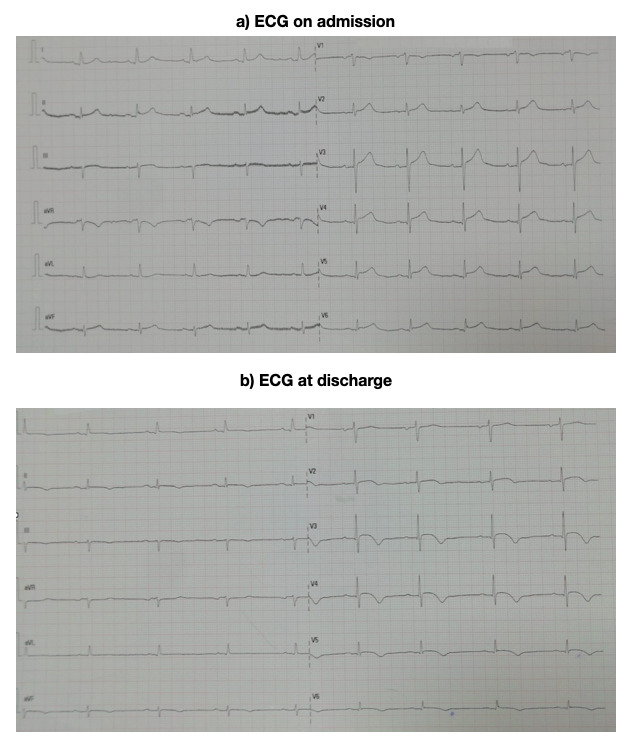
ECG at admission and at 24 hours. A) ECG on admission: sinus rhythm with a frequency of 61 beats per minute (bpm), the axis was 30°, the PR segment lasted for 0.19 milliseconds (ms), the QRS complex for 100 ms and there was a 0.1 mV elevation of the ST segment in the II and aVF derivations, and a 0.2 mV elevation in the V3, V4, V5 and V6 derivations. B) ECG at discharge: the elevation of the ST segment was still present and the T waves in the V3, V4, V5 and V6 derivations were beginning to become negative.

The patient is an 84-year-old woman with a medical history of hypertension and hypercholesterolemia on a type II angiotensin receptor antagonist (ARAII) and ezetimibe, who presented with an episode of mid-thoracic pain which radiated to her neck. The pain lasted for two hours and persisted as a slight to moderate discomfort until the patient finally went to the Emergency Room 24 hours later. The electrocardiogram (ECG) done at admission showed sinus rhythm of 61 beats per minute (bpm), with an axis of 30^o^, the PR segment of 0.19 milliseconds (ms), the QRS duration of 100 ms and a 0.1 mV elevation of the ST segment in II and aVF, and a 0.2 mV elevation in the V3, V4, V5 and V6 (Figure 1). The markers for myocardial injury were positive, with an ultrasensitive Tnl of 3832 ng/l (nanograms per liter). 

Since the patient was completely asymptomatic at this point, the case was interpreted as an anterolateral infarction in its subacute phase. Acetylsalicylic acid (ASA) and clopidogrel were administered and anticoagulant treatment with heparin of a low molecular weight was started. The patient was admitted to the Intensive Care Unit. 

Upon arrival, the patient was still asymptomatic, with a stable sinus rhythm and without a sign of cardiac failure. In the physical exploration an audible systolic heart murmur that radiated to the neck could be heard. 

**Figure 2:**
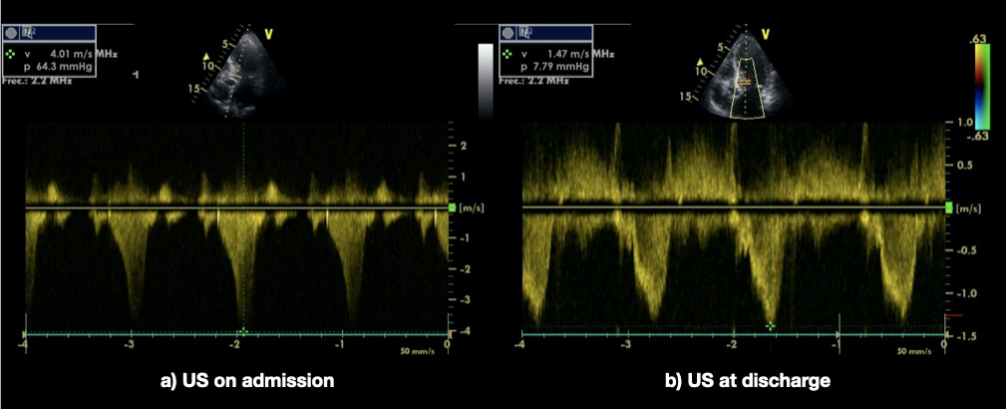
Dynamic systolic gradient in the exit tract of the left ventricle. At admission and 24 hours later. A) When it was studied using pulsed-doppler the spectral analysis had the characteristic keel shape of thesubvalvular dynamic stenosis. The gradient peak was 63 millimeters of mercury (mmHg). B) In the control echocardiography 24 hours later, there was a substantial decrease of the dynamic systolic gradient of the exit tract of the left ventricle, this was attributed to the beta-blocker treatment.

An advanced cardiac POCUS was performed (Video S1) and revealed the following:

- Non-dilated left ventricle, with an eccentric hypertrophy affecting the basal part of the interventricular septum.
- Severe hypokinesia of the apex and most apical segments of the anterior and lateral surfaces of the heart.
• Compensating hyperkinesia in the basal segments.
- Slightly depressed ejection fraction.
- Sclerosis and calcification of the mitral-aortic ring and of the valves.
- Abnormal pattern of relaxed diastolic filling.
- Moderate mitral regurgitation.
- Slight aortic regurgitation.
- In the study done with color-doppler, a turbulent flux appeared in the left ventricular outflow tract. When pulsed-doppler was applied, it had the characteristic knife shape of sub-valvular dynamic stenosis. The gradient peak was about 63 millimeters of mercury (mmHg). (Figure 2).
- In the two-dimensional image (2D) the movement of the anterior valve towards the septum during the systole (systolic anterior movement, SAM) could be clearly seen (Video S2).

**Figure 3:**
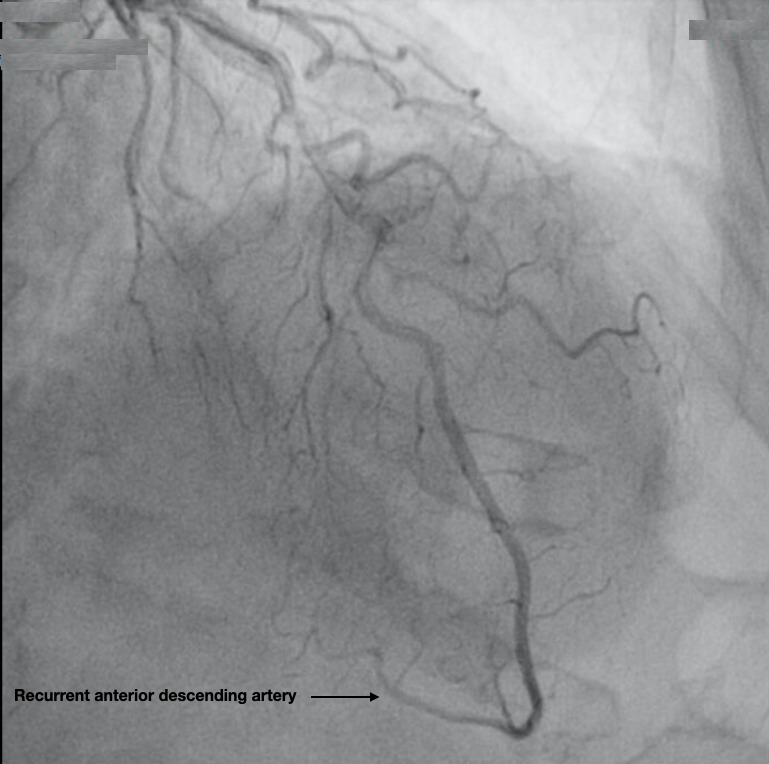
Redundant anterior descending artery. In some series of coronarography the anterior descending artery has shown a longer than average length and a longer recurrent diaphragmatic segment.

The maximum peak of ultrasensitive Tnl was reached at 5298 ng/l. In the ICU, anticoagulants, antiplatelets and beta-blockers (bisoprolol) were started. The following morning coronary angiography did not show any coronary lesions (Figure 3). Incidentally, the descending anterior artery was abnormally long, and wrapped around the apex and continued along the diaphragmatic surface. The diagnosis of Takotsubo syndrome was made, and the clopidogrel was stopped, while continuing the AAS and the bisoprolol. 

On the ECG done prior to discharge, 18 hours after the coronary angiography, elevation of the ST segment was still present while the T waves in V3, V4, V5 and V6 were beginning to become negative. The follow up echocardiography showed evidence of a substantial decrease of the dynamic systolic gradient of the left ventricular outflow tract (LVOT) which was attributed to the beta-blockers. 

## Conclusions

The Takotsubo syndrome was first described in 1990 by Sato et al.^1^, ^2^ and included in the American Heart Association’s (AHA) classification of myocardiopathies for the first time in 2006 under the name acquired primary stress cardiomyopathy 3.

The usual clinical presentation is indistinguishable from that of an acute coronary syndrome, since it produces the typical central thoracic pain, elevation of the ST segment in the ECG and an elevation of the markers for myocardial ischemia 4.

The echocardiographic image is very characteristic. The severe apical hypokinesia appears together with hyperkinesia in the basal segments which gives the heart the appearance of an inverted vase, the type traditional Japanese fishermen used to capture octopuses (tako-tsubo) 5. 

The hallmark of Takotsubo syndrome is that on coronary angiography the coronary arteries will show no significant lesions.

The etiology of the syndrome is still not definitively known. It has been observed that often the symptoms are triggered by a situation which causes emotional distress, and it has been postulated that the syndrome might be explained by the toxic effect of catecholamines amidst the excessive local sympathetic response 4. Given that Takotsubo predominantly affects postmenopausal women, lack of estrogens could be contributory since it is known that sex hormones exert an important influence over the sympathetic neurohormonal axis and coronary vasoreactivity 5.

On the echocardiography, a dynamic obstruction of the LVOT with systolic anterior movement of the mitral valve (SAM) can be seen in 20% of cases, generating an intraventricular gradient greater than 30 mmHg 6.

This gradient could be important in the genesis of the syndrome, because the obstruction elevates the intraventricular pressures of the apical area and diminishes myocardial perfusion (Video S2). In some series of coronarography the anterior descending artery has shown a longer than average length with a longer diaphragmatic course (Figure 3) 7.

After diagnosing Takotsubo and confirming it angiographically, antiplatelet treatment can be stopped, but beta-blockers must still be used, specially, in cases where an LVOT obstruction exists. 

Even though alterations in the left ventricular ejection fraction are characteristically transitory and functional recovery is the norm, some patients can present very severe cases of heart failure and even cardiogenic shock and death. 

For the emergency or ICU physician that uses POCUS, it is important to know the cardiac POCUS and echocardiographic characteristics of Takotsubo cardiomyopathy in order to raise the index of suspicion in patients presenting with chest pain. 

## Disclosures

None

## Supplementary Material

 Video S1 Point-of-care-ultrasound at admission. Eccentric hypertrophy of the left ventricle affecting the basal part of the septum. Severe hypokinesia of the apex. Compensating hyperkinesia in the basal segments. Abnormal pattern of relaxed diastolic filling. Moderate mitral regurgitation. Turbulent flux in the exit tract of the left ventricle with colour-doppler. Characteristic keel shape of subvalvular dynamic stenosis with pulsed-doppler. With high gradient peak (63 mmHg). Systolic Anterior Movement (SAM) of the mitral valve.

Video S2 Echocardiography at 24 hours with beta-blocker treatment. The control echocardiography showed evidence of a substantial decrease of the dynamic systolic gradient in the exit tract of the left ventricle (7.79 mmHg) and absence of SAM, which was attributed to the beta-blocker treatment.
